# Mapping amyotrophic lateral sclerosis lake risk factors across northern New England

**DOI:** 10.1186/1476-072X-13-1

**Published:** 2014-01-02

**Authors:** Nathan Torbick, Sarah Hession, Elijah Stommel, Tracie Caller

**Affiliations:** 1 Applied Geosolutions, 403 Kent Place, Newmarket, New Hampshire, USA; 2 Center for Statistical Training and Consulting, Michigan State University, 293 Farm Lane, Room 152B, East Lansing, Michigan, USA; 3 Department of Neurology, Dartmouth-Hitchcock Medical Center, 1 Medical Center Drive, Lebanon, New Hampshire, USA

**Keywords:** ALS, Cluster analysis, Lake water quality, Landsat, Logistic regression, Mapping, New England, Risk factors

## Abstract

**Background:**

Amyotrophic lateral sclerosis (ALS) is a progressive, fatal neurodegenerative disease with a lifetime risk of developing as 1 in 700. Despite many recent discoveries about the genetics of ALS, the etiology of sporadic ALS remains largely unknown with gene-environment interaction suspected as a driver. Water quality and the toxin beta methyl-amino-alanine produced by cyanobacteria are suspected environmental triggers. Our objective was to develop an eco-epidemiological modeling approach to characterize the spatial relationships between areas of higher than expected ALS incidence and lake water quality risk factors derived from satellite remote sensing as a surrogate marker of exposure.

**Methods:**

Our eco-epidemiological modeling approach began with implementing a spatial clustering analysis that was informed by local indicators of spatial autocorrelation to identify locations of normalized excess ALS counts at the census tract level across northern New England. Next, water quality data for all lakes over 6 hectares (n = 4,453) were generated using Landsat TM band ratio regression techniques calibrated with in situ lake sampling. Derived lake water quality risk maps included chlorophyll-a (Chl-a), Secchi depth (SD), and total nitrogen (TN). Finally, a spatially-aware logistic regression modeling approach was executed characterizing relationships between the derived lake water quality metrics and ALS hot spots.

**Results:**

Several distinct ALS hot spots were identified across the region. Remotely sensed lake water quality indicators were successfully derived; adjusted R^2^ values ranged between 0.62-0.88 for these indicators based on out-of-sample validation. Map products derived from these indicators represent the first wall-to-wall metrics of lake water quality across the region. Logistic regression modeling of ALS case membership in localized hot spots across the region, i.e., census tracts with higher than expected ALS counts, showed the following: increasing average SD within a radius of 30 km corresponds with a decrease in the odds of belonging to an ALS hot spot by 59%; increasing average TN within a radius of 30 km and average Chl-a concentration within a radius of 10 km correspond with increased odds of belonging to an ALS hot spot by 167% and 4%, respectively.

**Conclusions:**

The strengths of satellite remote sensing information can help overcome traditional field limitations and spatiotemporal data gaps to provide the public health community valuable exposure data. Geographic scale needs to be diligently considered when evaluating relationships among ecological processes, risk factors, and human health outcomes. Broadly, we found that poorer lake water quality was significantly associated with increased odds of belonging to an ALS cluster in the region. These findings support the hypothesis that sporadic ALS (sALS) can, in part, be triggered by environmental water-quality indicators and lake conditions that promote harmful algal blooms.

## Background

Amyotrophic lateral sclerosis (ALS) is a progressive, fatal neurodegenerative disease with a lifetime risk of development as 1 in 700 [1]. The pathologic hallmark of ALS is the selective death of motor neurons in the brain and spinal cord producing debilitating symptoms of progressive weakness, muscle wasting and spasticity. The average life expectancy of a person with ALS is two to five years from time of diagnosis with 5,600 new cases diagnosed per year in the USA [2]. Approximately 30,000 people in the United States have ALS at any one time. ALS is one of the most common neuromuscular diseases worldwide, and people of all races and ethnic backgrounds are affected. Incidence rates vary more widely in industrialized nations (an annual incidence rate of 0.2 to 2.4 per 100,000 population), compared to that in developing countries (1.5-2.0 per 100,000 per year) [3]. Overall, there is a slight male predominance (M:F ratio ~ 1.5:1).

Approximately two thirds of patients with typical ALS have a spinal form of the disease (limb onset) and undergo symptoms related to focal muscle weakness and wasting, where the symptoms may start either distally or proximally in the upper and lower limbs. Gradually, spasticity may develop in the weakened atrophic limbs, affecting manual dexterity and gait. Patients with bulbar onset ALS usually present with dysarthria and dysphagia for solid or liquids, and limbs symptoms can develop almost simultaneously with bulbar symptoms, and in the vast majority of cases will occur within 1–2 years. Paralysis is progressive and leads to death due to respiratory failure within 2–3 years for bulbar onset cases and 3–5 years for limb onset ALS cases.

The onset of ALS is age-related, with the highest incidence between 55 and 75 years of age [2,4]. As a consequence, the number of affected individuals should dramatically increase in future decades as the number of elderly persons rises. Mutations in genes underlying familial ALS (fALS) have been discovered in only 5-10% of the total population of ALS patients [5]. Approximately 90% of ALS cases have no known genetic cause; this group is commonly called sporadic ALS (sALS) [6,7]. Wijesekera and Leigh [8] provide a thorough review of ALS.

Despite many recent discoveries about the genetics of ALS, the etiology of sALS remains largely unknown. There is a broad scientific consensus that ALS is caused by gene-environment interactions [9]. It is most likely that sALS results from a combination of underlying genetic susceptibility and environmental exposure to one or more toxins, but much remains to be discovered. There has been no definitive incriminated environmental risk factor for ALS because not all studies of a particular environmental risk factor have been positive; tobacco is the only risk factor that seems to be consistently associated with ALS. The underlying genetic predisposing factors that render certain individuals more susceptible to a particular environmental toxin are also not well understood in ALS. The recently discovered hexanucleotide repeat expansion C9orf72 present in 7% of patients with sALS but only 0.2% of controls is likely to be one of those predisposing genetic factors [10].

Evidence has shown potential linkages between water quality, cyanobacteria, and ALS clusters [9]. Cyanobacteria are ubiquitous throughout all ecosystems and are particularly noxious when anthropogenic eutrophication of water bodies causes large concentrations to form “blooms”. Cyanobacteria are well-known to produce acute and chronic toxins that have human health implications, including cylindrospermopsins, lyngbyatoxins, anatoxins, lipopolysaccharide endotoxins and beta methyl-amino-alanine (BMAA) [11]. The 50- to 100-fold higher incidence of ALS documented amongst the Chamorro people of Guam implicated the cyanobacterial neurotoxin BMAA found in components of their diet [9,12-14].

The examination of other ecosystems has demonstrated the presence of BMAA in fish and crustaceans in the human food chain in Florida, Chesapeake Bay, France and Sweden [15-18]. BMAA has been demonstrated to be concentrated in the brains of ALS patients (but not controls) in Florida [19] and to be mis-incorporated into neuronal proteins via the L-serine tRNA-synthetase system [20-22]. Clusters of ALS have been reported near cyanobacterial bloom outbreaks in France, Japan, New Hampshire, and Wisconsin [23-27]. Caller et al. [28] shows a statistically significant 2.3-fold increased incidence of ALS in subjects residing within 0.5 miles of a New Hampshire lake that experienced cyanobacteria blooms. Potential routes of exposure include aerosolization, dermal contact, ingestion of water, and dietary exposure through the aquatic food web. The Baltic Sea suffers extensive cyanobacterial blooms generating BMAA as well as bottom-dwelling animals that contain BMAA and are a human food source [18].

### Water quality remote sensing

Assessment of lake conditions using traditional assessment methods can be costly and time consuming, severely limiting the temporal frequency and spatial coverage of these measurements. In the northeast US, typically no more than 15% of a State’s lakes are sampled using traditional field measurements. For instance, Maine has over 6,000 water bodies categorized as significant, yet rarely are more than 400 lakes sampled in a given year; typically New Hampshire samples about 150 of 950 lakes. Thus the use of operational satellite remote sensing has proven to be valuable technology to provide decision makers information on lake water quality, trends, and stressors. As the frequency and magnitude of Harmful Algal Blooms (HABs) have gained attention, many regions desire improved knowledge to begin to address public health threats and mitigate drivers. For example, best management practices, such as conservation tillage or storm water control, are being implemented in many regions to reduce flow of anthropogenic nutrients (e.g., nitrogen and phosphorus) contributing to algal growth, including cyanobacteria blooms that produce toxins such as BMAA.

The use of satellite remote sensing technologies has been used as an effective tool to derive information on lake water quality [29-41]. The choice sensor must consider a balance among spatial resolution, temporal overpass frequency, and spectral sensitivity as well as availability and cost. Cost efficient “wall-to-wall” maps of lake conditions over a large geographic region require the use of moderate resolution sensors, such as Landsat, due to the spatial configuration of small lakes and ponds [42]. A common application for inland lakes is to generate a Trophic Status Index [43] from Secchi depth (SD), a metric of clarity, or chlorophyll-a (Chl-a), a metric of quality and indictor of algae. In addition to lake state metrics, in situ lake measurements can also include biochemistry metrics such as total phosphorus (TP) or total nitrogen (TN), and ecohealth measures such as cyanobacteria density, diatom biovolume, or phytoplankton functional types.

Typically, a regression model using remotely sensed observations as independent variables is calibrated using a sample of in situ lake measurements to derive maps of lake quality. More analytical techniques that are sensitive to Inherent Optical Properties have been effective for single waterbodies or more advanced lake metrics such as Color Dissolved Organize Matter, cyanobacteria biovolume, or Non-Purgable Organic Carbon; however, analytical techniques for these metrics usually require remote sensing observations to be collected with narrow spectral channels with near-simultaneous in situ collection. Currently narrow spectral channels available on satellite platforms either have small footprints (e.g., EO-1) or relatively coarse spatial resolution (e.g., MERIS). Therefore, the use of Landsat to generate spatially comprehensive exposure metrics using chlorophyll-a as a surrogate of cyanobacteria along with complementing lake clarity (e.g., SD) and lake biochemistry (e.g., TN) is an effective approach for addressing public health concerns and identifying ‘hot spots’ [42].

### Eco-epidemiological modeling

Eco-epidemiology can be defined as the study of the relationships between ecological change and its influences on human health [44]. Epidemiology has traditionally focused on locations of disease, mortality and morbidity, and their distribution over space, trajectories of the disease, and causation. It has evolved over time, improving our understanding of infectious disease as well as risk factors. Eco-epidemiology is generally concerned with a broader spatial scale than that of traditional epidemiology, confronting human health risks on varying spatial scales, often synthesizing information on climate and landscape with changes in human behavior [44]. While many epidemiological or eco-epidemiological studies strive to understand infectious disease patterns, in which population dynamics of a disease vector are of concern [45-51], this study seeks to quantify exposures to lake risk factors and environmental toxins.

A variety of statistical models have been used to model disease risk. Messina et al. [47] conducted an aspatial multilevel logistic regression analysis, incorporating data at the level of individual and community, to estimate probability of malarial infection in the Democratic Republic of Congo; geographically-weighted regression was used to study the relationship between conflict and malaria prevalence. Loth et al. [50,51] used autologistic regression as a spatially explicit technique to model a dichotomous outcome (presence/absence of disease). However, shortcomings in this method have been identified by Dormann [52], resulting in biased estimates compared to aspatial logistic regression and underestimation of the effect of environmental variables. Paul et al. [49] utilized hierarchical Bayesian modeling to compute area-specific relative risk estimates while considering spatial interactions through a spatial smoothing based on a Gaussian auto-regressive model. Goovaerts [53] describes the application of poisson kriging to map mortality risk, and geographically-weighted regression to account for varying regression coefficients over space. Following approaches summarized above the characterization of relationship linkages, or stressor – response patterns, between landscapes and public health outcomes is feasible. Further, eco-epidemiological modeling can help identify valuable ecosystem services that support public health, and in turn be used to promote management practices and policies that foster sustainable resource use.

## Results and discussion

### ALS Hot spots

**Figure 1:**
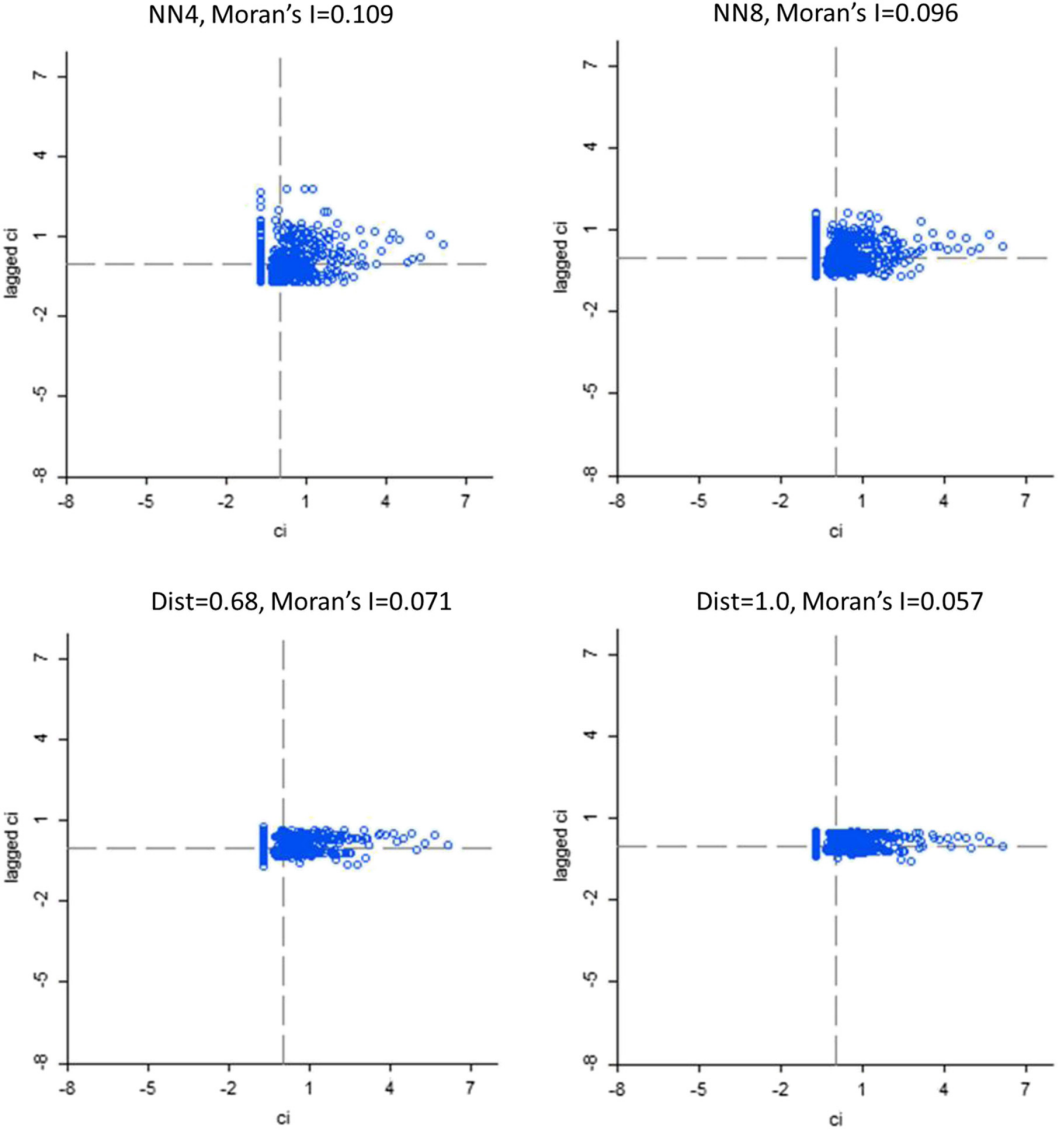
**Global Moran’s I, a global measure of spatial autocorrelation, calculated using tract-level aggregated ALS case data.** Results are shown for 4 and 8 nearest neighbors (NN) and distance bands of 0.68 DD and 1.0 DD. The highest level of spatial autocorrelation is observed at a scale of 4 nearest neighbors.

Normalized excess case counts were estimated for each census tract within the study area. These results were evaluated for global spatial autocorrelation using the global Moran’s I statistic at a variety of nearest-neighbor configurations (Figure 1) to identify an optimal scale for estimating spatial autocorrelation. A configuration of 4 nearest neighbors (i.e., 4 nearest census tracts) was selected for further analysis. In other words, spatial autocorrelation among the excess case counts was found to be highest when considering the four nearest census tracts rather than considering a larger number of census tracts or larger distances.

**Figure 2:**
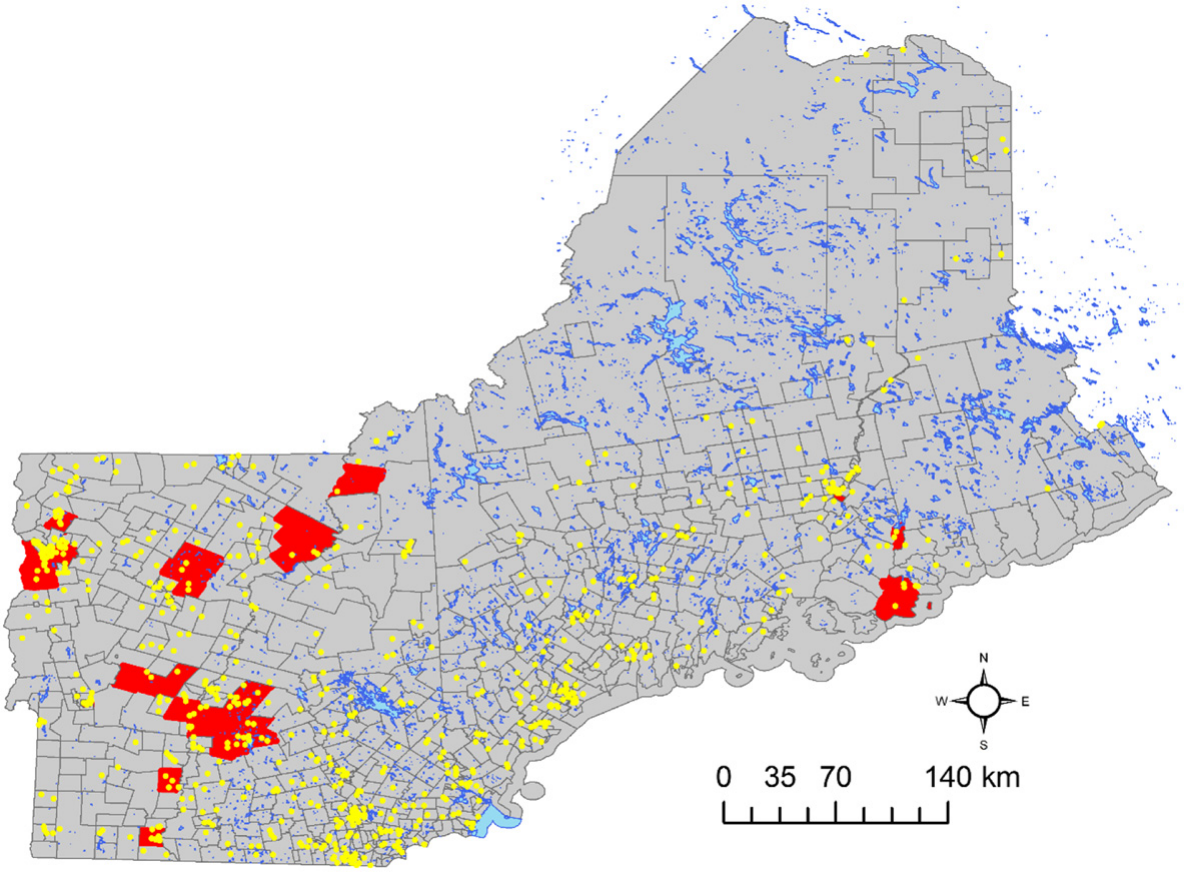
**Tract scale ALS hot spots (red) representing census tracts with statistically significant higher than expected normalized ALS cases with yellow dots showing ALS patients and blue lakes across northern New England.** Yellow dots have been geomasked as to not show exact locations to protect privacy.

Local indicators of spatial autocorrelation were calculated to identify localized clustering of census tracts with higher incidence or ‘hot spots’. Results are mapped by census tract in Figure 2. Census tracts shown in dark red indicate significant positive spatial autocorrelation among census tracts. Large clusters of census tracts identified in red are located around Burlington, VT, adjacent to Lake Champlain which undergoes periodic harmful algal blooms. Another large cluster is identified in the greater Hanover region, which is a short distance from DHMC. Individual hot spot census tracks are also located along coastlines, in higher elevation mountain regions, within populated suburban communities, and in largely rural tracts. This indicates a dispersed geography of sALS hot spots in New England. This cluster analysis expands upon [24,28] with additional methodologies that reflect similar patterns of clustering across the northeast, USA.

### Mapping lake water quality

**Figure 3:**
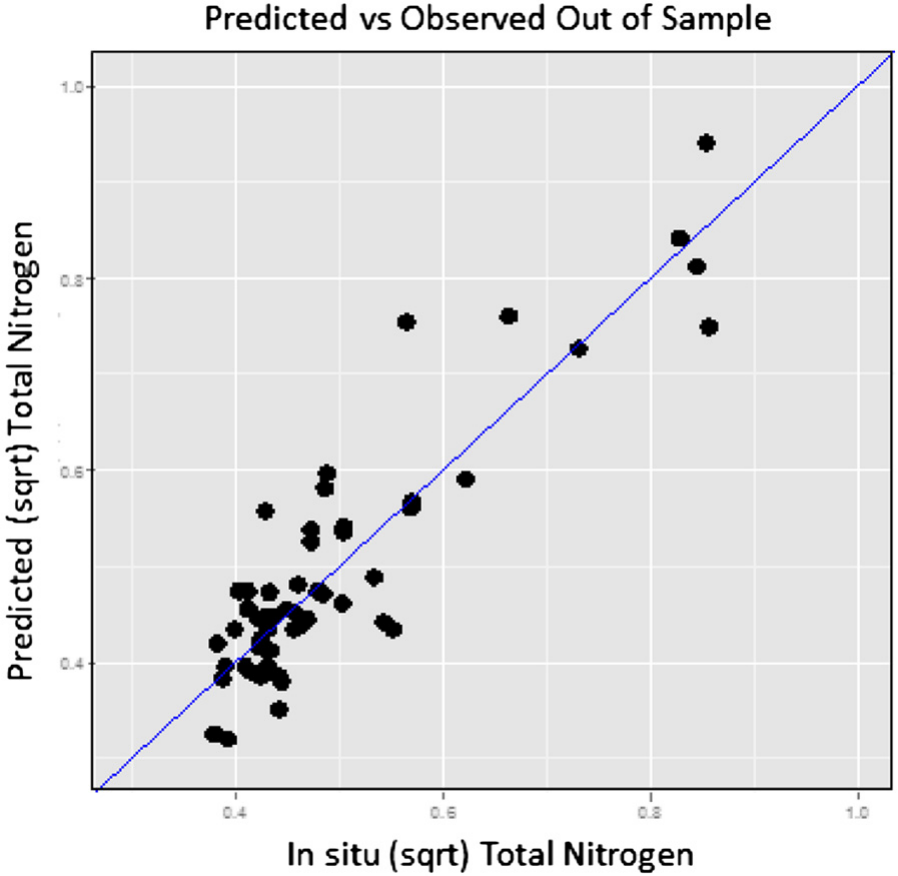
Example landsat total nitrogen algorithm with out of sample predictions and in situ samples with 1:1 line included.

Remotely sensed water quality metrics were generated for Chl-a, SD, and TN. The Landsat band ratio regression approach had adjusted R^2^ of 0.62, 0.88, and 0.79 for Chl-a, SD, and TN, respectively. Figure 3 shows the predicted out-of-sample results for TN for lakes across Maine path 12 indicating a rigorous algorithm with generally low scatter along the 1:1 line. Considering the range of lake states, number of path-row combinations, and large geographic region these are satisfactory adjusted R^2^ results compared to similar applications (e.g., [37,41,42]). The Chl-a algorithm might be improved with more concordant in situ sampling on Color Dissolved Organic Matter samples that potentially creates signal noise within the broad Landsat spectral channels, especially in lakes with lower production. A more complex chl-a model did achieve a higher significant adjusted R^2^; however, the F-statistic and AIC values, along with a lack of explanatory power of significant bands, did not support the case to replace the model executed in this application. The lake metrics selected for use in this application are effective and well-established indicators of lake water quality, were not highly correlated thus yielding independent information, and can be accurately mapped with satellite remote sensing technologies.

Water quality in northern New England is geographically variable, even among lakes and watersheds. Evident in the maps is that many of the watersheds with poorer water quality conditions are associated with areas and corridors of higher development and resource utilization. The majority (82%) of lakes over 6 hectares in Maine, New Hampshire, and Vermont were categorized as oligotrophic and mesotrophic according to a derived trophic status index [43], indicating relatively healthy waters. Approximately 13% were classified as eutrophic waters with eutrophic lakes located across the region. Of the 4,453 lakes mapped, 185 were characterized as hypereutrophic or nutrient-rich lakes. It is not uncommon for hypereutrophic lakes to undergo frequent and intense algal blooms. We note that large lakes can have substantial variability often with bays, coastlines, and inlets having elevated biological and physiochemical indicators. For example, overall, Lake Winnipesauke is considered an average oligotrophic lake; however, Alton and Wolfeboro Bays and Chestnut Cove have blooms and relatively higher concentrations of cyanobacteria algae. So, a recreational-use boater using the entire lake might have a completely different risk profile compared to an individual swimming along a beach located in a bay with elevated indicators.

**Figure 4:**
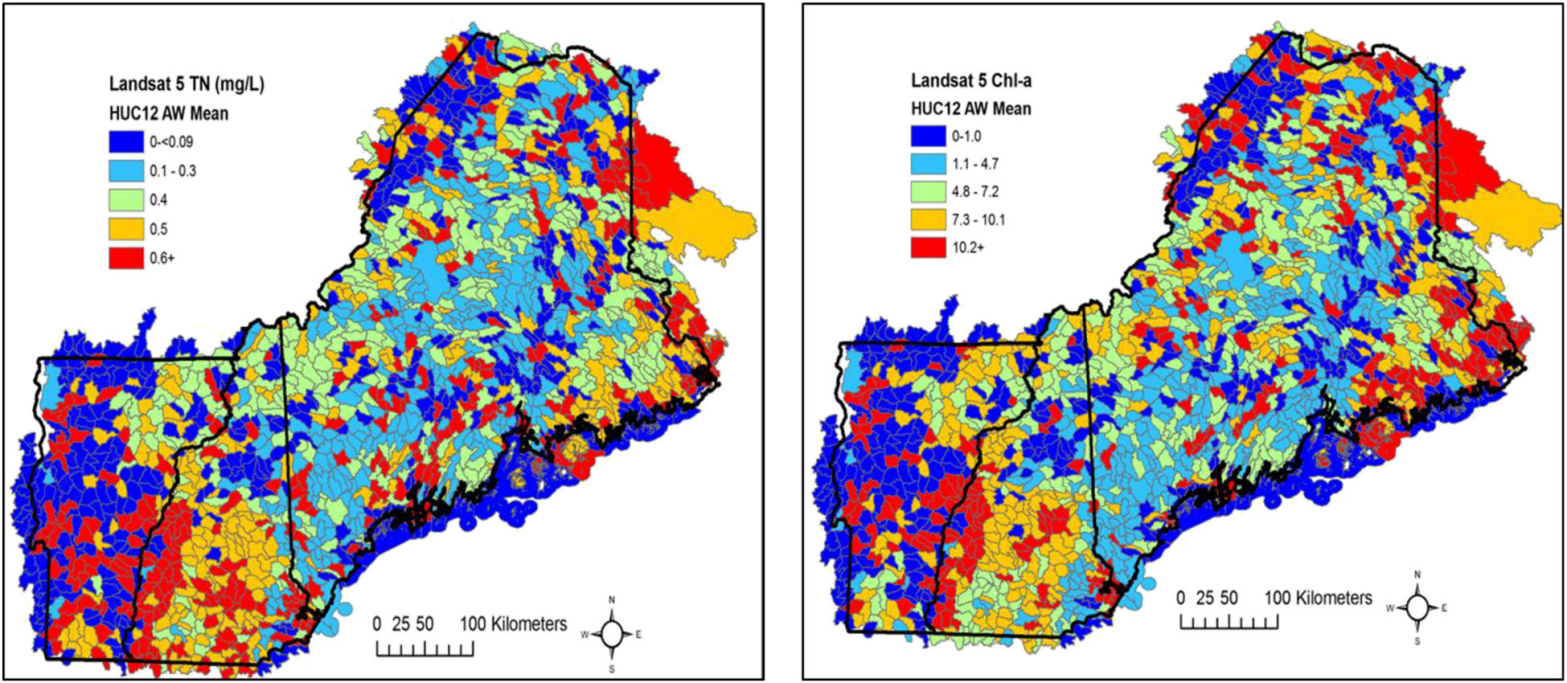
Area-weighted mean total nitrogen and chlorophyll-a concentration at the watershed scale.

When considering ecological processes and patterns of exposure to varying levels of water quality, it is important to carefully consider geographic scales. In some watersheds a few extreme lakes that are large in size can influence metrics. Further, the possible breadth of ‘geographic’ metrics- such as driving distance to lake, problematic beaches, or drinking water source -is daunting so we diligently considered a range of metrics while taking into account the availability of spatial data and objective of the research. Therefore, we generated a suite of lake metrics at a range of scales to evaluate as potential risk factors. Figure 4 shows average, lake area-weighted TN and Chl-a at the watershed (HUC12) scale. Euclidean distance represents a distance a population, or ALS patient, lives from a water body; we focused on exposures within 10 and 30 km which were chosen based on the size and distribution of lakes, census tracts, and road networks. We also generated a suite of geographic measures such as the number of lakes within 10 and 30 km of an ALS case and minimum distance to a lake.. We systematically summarized lake data at these given scales and tested these metrics within our modeling framework.

More direct measures of cyanobacteria density and biovolume are required to generate operational cyanobacteria metrics from Landsat as these have remained a challenge due to broad spectral bands and lack of data availability. With more intensive cyanobacteria sampling, Landsat-derived cyanobacteria indices (e.g., [40,42]) might become more commonplace for small lakes across expansive geography. The use of hyperspectral data, more direct analytical techniques sensitive to Inherent Optical Properties (IOP), or platforms with narrow spectral channels (i.e., MERIS) have shown promise to more precisely map attributes (e.g., [54-56]); however, hyperspectral data tend to be expensive and limited, analytical techniques are challenging to apply in diverse Case II waters, and sensors such as MERIS (no longer operational) do not have adequate spatial resolution for smaller inland lakes. With 30 m spatial resolution and spectral coverage in the visible and near infrared wavelength domains, Landsat has the ability to map lake metrics for waterbodies as small as 6 hectares to provide wall to wall exposure data, which is required for a comprehensive evaluation of lakes as potential ALS risk factors.

It is feasible that the amount of calibration data needed for operational Landsat water quality metrics will become less of an obstacle as the number of citizen scientists and volunteer monitoring groups continue to grow. However, teasing out phytoplankton functional types or measuring cyanobacteria biovolume requires expertise and laboratory facilities. Thus the continued education of the public on lake quality indicators as well as support for monitoring programs is essential if we are to achieve cost efficient, operational cyanobacteria risk maps for small lakes. The literature ranges in the use of terms such as phytoplankton functional types, blue green algae, toxin producing cyanobacteria, or harmful algal blooms (eukaryotes) to describe lake events and outcomes. In this research application we highlight that varying toxin producing cyanobacteria (prokaryotes) can produce BMAA at different magnitudes and frequencies. Further, the stressor response behavior between nutrients (e.g., TN and total phosphorus), productivity and algae growth, and many biological and physicochemical indicators are interlinked with complex and non-linear patterns that can vary by lake state.

### Assessing lake water quality as risk factors

Logistic regression modeling was completed to evaluate for significant relationships between ALS case membership in census tracts identified as hot spots, and a number of potential predictors or risk factors of hot spots. Figure 2 shows the approximate locations of the individual ALS cases with red census tracts highlighting areas of statistically significant, higher than expected counts, thus depicting the geographic distribution of the dependent variable used in our eco-epidemiological model. For modeling purposes, the dependent variable was coded 1 if an ALS case lived within a census tract identified as part of a hot spot, 0 if not. When the dependent variable is coded this way for a logistic regression analysis, predictions can be made that correspond to the probability of hot spot membership. We found that of the 754 ALS cases included in the model, 83 (11%) belong to hot spots across the region in 34 hot spots.

Table 1 summarizes the independent variables, or hot spot risk factors, and the final subset of models considered, illustrating selection of scale for the water quality variables included in the final logistic regression model. Ultimately, no geographic variables were included in the analysis. Numbers of lakes within a radius of 10 or 30 km were excluded due to high multicollinearity with other risk factors. Minimum distance to a lake was excluded due to difficulty in interpreting results. The difficulties in interpreting the relationship between minimum distance to a lake and risk of belonging to an ALS hot spot likely arose due to multicollinearity with other risk variables, or methods in estimating the minimum distance (e.g., use of distance to lake centroid versus distance to nearest coastal point). Additional work is necessary to further evaluate the influence of distance from ALS cases to lakes. It is possible that other geographic factors such as driving distance to beach, location of water treatment plants, or location along hydrological network might influence the model, but these other types of geographic parameters were not included in this initial study exploring relationships between lake water quality and ALS cases. The first set of models shown on Table 1 that include one water quality parameter each was used to identify optimal scale for each parameter. Based on lower AIC values, scales of 10 km, 30 km, and 30 km were selected for Chl-a, SD, and TN, respectively. Including two water quality parameters generally improved model fit. Including all three water quality parameters (model 9) yielded the lowest AIC value.

**Table 1:** Summary of eco-epidemiological models and AIC rankings for predicting ALS hot spot membership

	**AIC**	**Notes**
Models with 1 WQ parameter, varying scales		
1) CHLA30 km	502.43	
2) CHLA10 km	483.63	Best scale is 10 km
3) SD30 km	463.53	Best scale is 30 km; best single parameter
4) SD10 km	501.65	
5) TN30 km	491.16	Best scale is 30 km
6) TN10 km	508.61	
Models with 2 WQ parameters, varying scales		
7) SD30 km + CHLA10 km	461.73	
8) SD30 km + TN30 km	453.62	
Model with 3 WQ parameters at best scales		
9) SD30 km + TN30 km + CHLA10 km	452.36	Best WQ and GEO model

The final selected model is provided in Table 2. As shown in Table 2, SD (30 km) and TN (30 km) are significant predictors of ALS hot spots at a 5% level of significance with Chl-a (10 km) a significant predictor of ALS hot spots at a 10% level of significance (*p* = 0.0678). In logistic regression, the regression coefficients (β) are not easily interpreted. However, odds ratios (OR) can be calculated as exp(β), and can be interpreted as a measure of association between a predictor, or potential risk factor, and an outcome. The OR represents the increase or decrease in the odds that an outcome will occur given a unit increase in the potential risk factor. The ORs for TN (30 km) and Chl-a (10 km) are greater than 1, indicating that increasing values of these variables are associated with higher odds of belonging to an ALS hot spot (Table 2). Specifically, each unit increase in TN is associated with a 2.4 times increase in the odds of belonging to an ALS hot spot. A 5% increase in odds is associated with every unit increase in Chl-a. The odds ratio for SD is less than 1, indicating that the odds of belonging to an ALS hot spot decrease with increasing values for SD: the likelihood of belonging to an ALS hot spot is approximately 59% lower for each unit increase in SD (i.e., for increasing water clarity). This analysis illustrates, in general, that poorer water quality is associated with higher odds of belonging to an ALS hot spot.

**Table 2:** Summary of selected logistic regression model for hot spot membership

**Independent variables**	**Regression coefficient**	**Std. error**	**z- value**	**p-value**	**OR**	**95% confidence interval**	
						**LCL**	**UCL**
(Intercept)	−0.066	0.972	−0.07	0.9461	0.94	0.142	6.498
SD30 km	−0.921	0.231	−3.99	0.0001	0.40	0.249	0.614
TN30 km	0.884	0.262	3.38	0.0007	2.42	1.460	4.124
CHLA10 km	0.045	0.024	1.83	0.0678	1.05	0.996	1.098

p-values are interpreted as follows: < 0.10 significant at the 90% significance level; < 0.05 significant at the 95% significance level; < 0.01 significant at the 99% significance level. Odds ratio (OR) represents the odds of belonging to a hot spot: OR > 1 indicates increasing odds of belonging to a hot spot for each unit increase in the independent variable, OR <1 indicates decreasing odds of belonging to a hot spot for each unit increase in the independent variable. Lower confidence limits (LCL) and upper confidence limits (UCLs) are provided.

We note that, in this study, TN was a stronger predictor of ALS hot spot membership than Chl-a concentration. It is well established that nitrogen, along with phosphorus, are drivers of cyanobacteria and harmful algal blooms in inland freshwater lakes [57]. The lower R^2^ and thus greater uncertainty of the Chl-a algorithm relative to SD and TN models also potentially diminish the role of Chl-a as a surrogate for BMAA exposure. The non-linear relationship between Chl-a concentration and cyanobacteria biovolume [42] will require additional modeling and field data to confirm this hypothesis.

**Figure 5:**
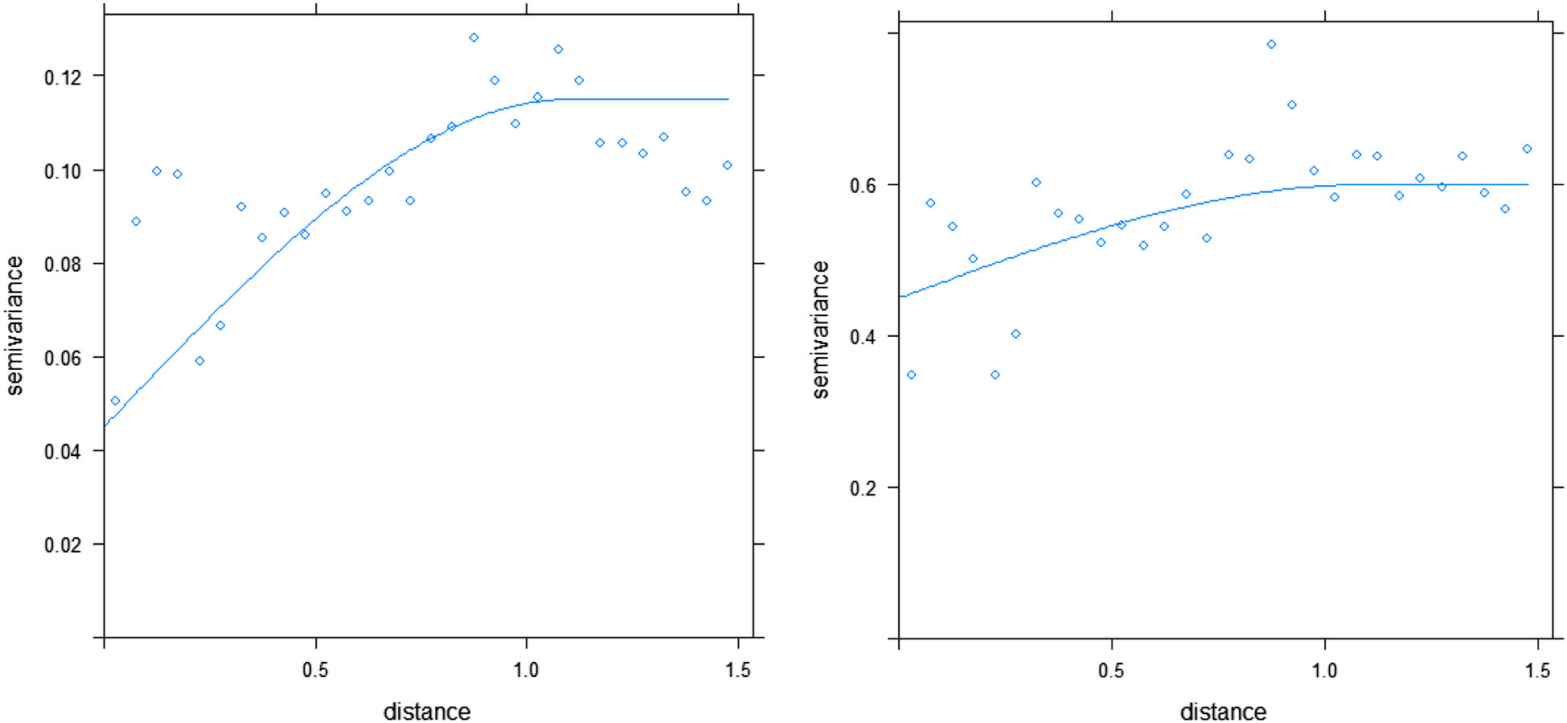
Variogram of dependent variable (hot spot membership) (left) and variogram of residuals from logistic regression model (right).

As previously noted, an aspatial logistic regression analysis was conducted to evaluate for significant relationships between ALS case membership in hot spots and potential risk factors of hot spots. An autologistic regression analysis was not used due to expected bias in the resulting regression coefficients; this bias would consistently underestimate the effect of the environmental variable or risk factor in the model compared to a non-spatial logistic regression [52]. Consequently, an aspatial logistic regression model was fit initially, and residuals were evaluated for spatial autocorrelation. As shown in Figure 5, a large portion of the spatial autocorrelation in hot spot membership is explained by use of spatially varying risk factors in the aspatial logistic regression model. This is illustrated when comparing the semi-variogram for the dependent variable, hot spot membership (left), to the semi-variogram for the final model residuals (right). The nugget to sill ratio is much smaller for the hot spot membership semi-variogram than the residuals semi-variogram. A small nugget to sill ratio indicates that the dependent variable, hot spot membership, is much less variable at a small spatial scale than at larger spatial scales, indicating the presence of spatial autocorrelation. Conversely, the difference between the nugget and sill is very small for the model residuals, indicating that there is little added variability in the residuals as spatial scales increase. Consequently, the aspatial logistic regression model adequately modeled a spatially varying dependent variable with spatially varying independent variables, leaving little spatial autocorrelation in the residuals.

This study successfully used an aspatial logistical regression model in a spatially-aware context to develop an eco-epidemiological framework for predicting ALS hot spot membership based on lake water-quality indicators. This approach, which quantifies exposures to lake risk factors and environmental toxins, can be used to evaluate the environmental health of valuable ecosystem services that link to public health, and in turn be used to promote management practices and policies that foster sustainable resource use.

**Figure 6:**
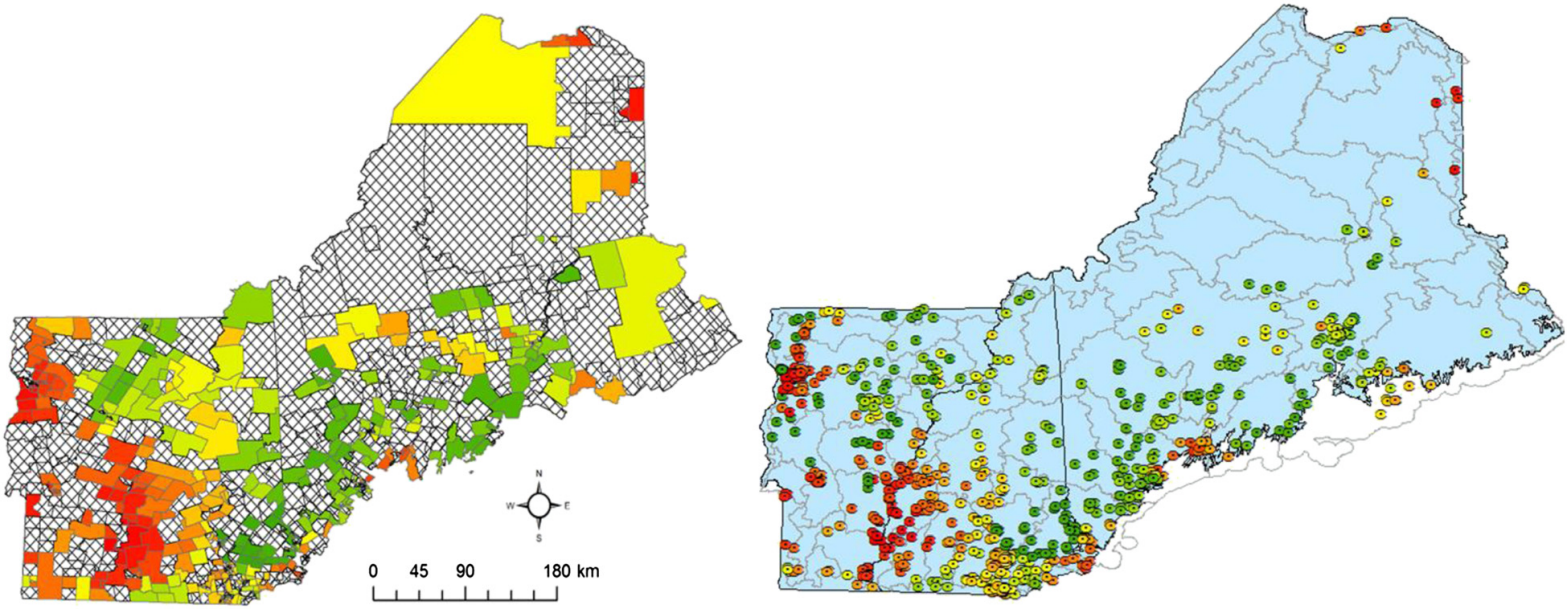
**Risk of belonging to a localized cluster of higher than expected ALS counts based on eco-epidemiological model by census tract (left) and ALS patient location (right) scales (risk displayed as low (green) to high (red)).**

Map of the probability of belonging to an ALS hot spot was derived at the census tract scale and the scale of individual ALS case from the final eco-epidemiological model and lake water quality satellite remote sensing metrics (Figure 6). These maps will be used to guide additional research for higher resolution analyses and generating long term exposure data from image archives.

### Conclusions, limitations, and future work

This research demonstrates a significant association between remotely sensed indicators of lake water quality and the odds of an ALS case belonging to an ALS hot spot, i.e., a census tract with higher than expected ALS counts. In general, poorer water quality was significantly associated with higher risk of belonging to an ALS hot spot. The research findings support the *hypothesis* that sALS can be triggered by environmental lake water quality and lake conditions that promote HABs and increases in cyanobacteria. This study found that significant predictors of ALS hot spot membership included Chl-a which served as a surrogate for cyanobacteria growth, TN a direct driver of algae growth, and SD, a broad measure of water clarity. To the best of our knowledge this work represents one of the first studies to spatially link residential location, sALS cases, and inland lake water quality. The results emphasize the valuable role of fresh water lakes in providing ecosystem services that influence public health.

We recognize and highlight there are other potential risk factors and that some of these risk factors potentially interact or reside in lakes that undergo HABs. The array of environmental and occupational toxins that have been implicated include several other exposure pathways that were not included in this study. For example, heavy metals lead and mercury [58-61], selenium [62], and agricultural pesticides [63,64] have all been proposed as influential drivers of sALS. Lifestyle factors and other toxins implicated also include tobacco [65,66], military service [67,68], and head injuries [69-71].

We aim to improve upon the remote sensing algorithms and include additional in situ lake sampling for cyanobacteria biovolume and density in future work. Collection of additional field data will reduce uncertainty in satellite remote sensing algorithms and improve the accuracy and precision of mapping risk factors. Our eco-epidemiological model will benefit from increased precision in risk factors, improving our understanding of the relationship between these factors and membership in sALS clusters. We also hope to expand our eco-epidemiological model and spatial data analysis to include additional geographic variables that summarize patterns of exposure to inland lakes and further refine our analysis of spatial scale, i.e., looking at watershed histories, landscape pattern metrics of agriculture, and road distances to beaches. Adding temporal components that assess trends in lakes, clusters of sALS, and the influence of other forcings (e.g., climate change), will enhance the work and help address the etiology of ALS. Patient questionnaires detailing exposure history are being compiled which will shed more insight on the potential role of BMAA in driving sALS patterns in NNE. To the best of our knowledge this work represents one of the first studies to spatially link residential location, ALS cases, and inland lake water quality. Potentially, the approach outlined in this research is applicable to other neurodegenerative diseases such as Parkinson’s Disease; however, more work is required to evaluate spatial location, exposure history, and toxins as a driver. Overall, we emphasize the value of the holistic approach using multiple lake quality attributes and the role of freshwater lakes in supporting human health.

## Methods

### ALS patient data

Data on ALS cases were derived from an existing database created by the ALS Center at Dartmouth Hitchcock Medical Center (DHMC). Records from DHMC, the Muscular Dystrophy Association of Northern New England, and surveys were searched to identify cases of ALS diagnosed between January 1997 and October 2009. When possible, we confirmed accuracy of diagnosis, year of diagnosis, demographic history of patients identified by review of medical records, the Social Security Death Index, obituaries, and data supplemented from questionnaires. The questionnaire assessed all current and prior dwelling locations, medical history, occupational history, and environmental exposures. For each identified case, we collected the age at diagnosis, year of diagnosis, family history, and dwelling address(s). Based on this information, we created a geocoded spatial database with more than 800 ALS patients across New Hampshire, Vermont, and Maine, or northern New England (USA). Data collection and study methods were approved and overseen by the Committee for the Protection of Human Subjects (#20332) at Dartmouth college and the National Institute of Health.

### Remote sensing

Landsat Thematic Mapper (TM) observations for Worldwide Reference System (WRS) path rows 10–12/27–30 were obtained from Earth Explorer (earthexplorer.usgs.gov/). Imagery was assessed for cloud coverage and quality with target acquisition dates during late summer to correspond to seasonal lake ecology dynamics. Overpass dates for the years 2009 and 2010 for Day Of Year 242, 244, and 248 were used in this research application to cover the entire study area. Raw data were resampled using cubic convolution algorithms, orthorectified, and delivered in the Universal Transverse Mercator (UTM) projection as geotiffs. The TM instrument collects spectral information in seven bands across the visible (0.45-.69 *u*m), near- (nir: 0.76-0.90 *u*m) and shortwave-infrared (swir: 1.55-1.75, 2.09-2.35 *u*m), and thermal (10.40-12.50 *u*m) domains of the spectrum at 16-day repeat intervals. Imagery was preprocessed to water leaving radiance and lake mapping approach followed methods detailed by Torbick et al. [42].

In situ lake data from regional government agencies were aggregated into a Geographic Information System (GIS). We focused on measurements of SD, TN, and Chl-a. SD is a common measure of water clarity that is easy to interpret and track; Chl-a is a common measure of algae volume; and TN is an important nutrient that drives trophic states and cyanobacteria blooms in the region thus providing a key metric of loading and lake chemistry. These metrics were chosen because they were not highly correlated, are all well-established water quality assessment parameters, and can be effectively mapped with remote sensing data. Lake vector polygons obtained from local government agencies were then used to extract the remote sensing information to generate lake average data for each band. A 60 m buffer was applied to avoid any mixed pixels or noisy coastlines. Lakes smaller than six hectares were excluded due to spatial constraints and sample size, which resulted in 4,453 total lakes studied across northern New England.

Once a database with concordant satellite data and lake measurements was constructed we executed an initial data mining routine. An exhaustive forward and backward stepwise linear regression using ordinary least squares was performed in the R statistical software [72] using an efficient branch-and-bound approach. This step was performed to highlight potential variables to use for model building. A correlation matrix was applied to reduce redundant variables. Following data mining, strategic linear regressions were conducted using variables shown to have spectral relationships with water quality properties in previous studies. The number of lakes used for model development varied depending on aggregation scheme (e.g., scene, path, date, month, etc.…) and all scales were mined following a ‘big data’ approach. We examined the behavior of linear models using strategic bands and band ratios using F-statistics, adjusted R^2^, significance values, root mean squared error (RMSE), normal probability (Q-Q) plots, and Cook’s Distance. Akaike information criterion (AIC; [73]) was then applied to a subset of strategic models to further help compare models. We ended up using a 7 day threshold (<7) between date of in situ lake sampling and satellite overpass for our final models; however, the models were relatively rigorous whether smaller (<3 days) and larger (12 days) temporal windows were applied. In addition to the statistical results we considered behavior of lake phenology, image availability and quality, and atmospheric conditions to determine 7 days was optimal for this research. After considering models we performed n-folds cross validation to assess the performance using out of sample results for each of the three lake attributes. Lake water quality mapping methods follow the approach detailed in Torbick et al. [42].

### Cluster analyses

Spatial analysis was performed at the census tract level to identify census tracts or clusters of census tracts with higher than expected numbers of ALS patients. First, an expected ALS case count was calculated for each census tract using published ALS data in the United States [74] after adjusting for local population density and sex distribution. Noonan et al. [74] reported an overall mortality rate of 1.82 per 100,000 due to motor neuron disease from 1994 to 1998. Direct age-adjusted [75] gender-specific rates of 2.17 (male) and 1.48 (female) are also reported for the same time period. The gender-specific rates were applied to the total counts of males and females by census tract and summed across gender to estimate a total expected count by census tract. The expected counts were then used to estimate the normalized excess case count (*c*_*i*_) for each census tract as follows [16]:

$$c_{i}=\frac{\left(o_{i}-e_{i}\right)}{e_{i}}$$

where *o*_*i*_ represents the number of observed ALS cases per census tract and *e*_*i*_ represents the expected number of cases.

Normalized excess case counts were evaluated for global spatial autocorrelation using the global Moran’s I statistic [76]. A variety of nearest-neighbor configurations was used to calculate spatial weights and identify an optimal configuration for measuring spatial autocorrelation. A limitation of the global Moran’s I statistic is that it is calculated with the assumption that any clustering of results occurs on a broad scale over the study area, rather than localized clustering. Consequently, local indicators of spatial autocorrelation (LISA; [76,77]) were calculated to identify localized clustering of census tracts with higher incidence. Census tracts with significantly higher incidence, which are defined here as hot spots, were identified as statistically significant when z-scores corresponding to the LISA statistics were greater than 2.58 with p-values less than 0.01. Evaluation of spatial autocorrelation and cluster analyses were conducted using GeoDa [78].

### Logistic regression

Logistic regression modeling [79,80] was completed at the case level to evaluate for relationships between individual ALS case membership in census tracts with higher-than-expected ALS counts, or hot spots, and potential risk factors including water quality indicators based on the lake risk maps generated from the remote sensing data (Table 3). The basic form of the logistic regression model is:

$$F\left(Y_{i}\right)=\beta_{0}+\beta_{1}X_{1i}+\beta_{2}X_{2i}+\ldots +\beta_{k}X_{\mathrm{ki}}+\epsilon_{i}$$

where *Y*_*i*_ represents the dichotomous dependent variable (1 = ALS case belongs to a hot spot, 0 = ALS case does not belong to a hot spot); *X*_1_, …, *X*_*k*_ represent the independent variables or potential risk factors; *F*(*Y*_*i*_) is a logit link function to transform the binary dependent variable to the appropriate scale for estimation of regression coefficients (*β*_1_, …, *β*_*k*_); and *ϵ*_*i*_ represents random residual errors*.* Regression coefficients are exponentiated, *e*^(*β*)^, for interpretation as odds ratios.

**Table 3:** Independent variables, or hot spot risk factors, considered in the analysis are divided in two categories: water quality parameters or geographic variables

**Variable**	**Scale(s)**	**Notes**
**Water quality parameters**	10, 30 km	All 754 ALS cases had at least 1 lake within 30 km; 709 ALS cases had at least one lake within 10 km.
Chlorophyll-a		
Secchi depth		
Total nitrogen		
**Geographic variables**	10, 30 km	All 754 ALS cases had at least 1 lake within 30 km; 709 ALS cases had at least one lake within 10 km.
Minimum distance to lake		
Number of lakes within given radius		

We strategically tested multiple scales by generating averages of lake water quality by fixed radii of 10 km and 30 km. Geographic variables considered included minimum distance to lake and number of lakes within various spatial scales. A strategic set of logistic regression models was evaluated to identify significant risk factors and scales in predicting hot spot membership. Initially, models that incorporated the variables that did not vary by scale were fit and compared to identify a set of base variables for the model. Scale-dependent variables were then added to the base model one at a time, fitting a separate model for each scale, to identify the scale for each variable that most improved model fit. A final set of models that combined the scale-dependent variables at their optimal scales was then evaluated. All models were evaluated using variance inflation factors (VIFs; [81]) to evaluate for multicollinearity and AIC [73] as a measure of model fit. Ultimately, the best fitting and most interpretable models were identified to create risk maps. Logistic regression modeling was completed using R statistical software [72], including the packages “gstat” [82] and “car” [83].

Researchers have used autologistic regression models to address issues of spatial autocorrelation in evaluating the relationship between risk factors and disease occurrence [50,51]. Although hot spot membership is spatially autocorrelated, we chose not to use an autologistic model due to expected bias in the resulting regression coefficients [52]. As stated by Dormann, autologistic regression models consistently underestimate the effect of the environmental variable in the model and give biased estimates compared to a non-spatial logistic regression. Rather, an aspatial logistic regression model was fit initially, and residuals were evaluated for spatial autocorrelation. This approach was deemed most appropriate for this study because the potential risk factors considered in the logistic regression model also vary over space.

## Abbreviations

ATSDR: Agency for toxic substances and disease registry; AIC: Akaike information criterion; ALS: Amyotrophic lateral sclerosis; BMAA: Beta methyl-amino-alanine; CDC: Centers for disease control and prevention; Chl-a: Chlorophyll-a; DHMC: Dartmouth Hitchcock medical center; fALS: Familial amyotrophic lateral sclerosis; GIS: Geographic information systems; HAB: Harmful algal bloom; HUC: Hydrological unit code; KM: Kilometer; LISA: Local indicators of spatial autocorrelation; OR: Odds ratio; RMSE: Root mean square error; SD: Secchi depth; sALS: Amyotrophic lateral sclerosis; TM: Thematic mapper; TN: Total nitrogen; TP: Total phosphorus; UTM: Universal transverse mercator; VIF: Variance inflation factors; WRS: Worldwide reference system.

## Competing interests

The authors declare that they have no competing interests.

## Authors’ contribution

NT led remote sensing, project integration, and co-led geospatial risk modeling. ES and TC led the human health data. SH co-led the geostatistical modeling. All authors read and approved the final manuscript.
